# Prevention and Control of Porcine Epidemic Diarrhea: The Development of Recombination-Resistant Live Attenuated Vaccines

**DOI:** 10.3390/v14061317

**Published:** 2022-06-16

**Authors:** Xiaoyu Niu, Qiuhong Wang

**Affiliations:** 1 Center for Food Animal Health, Department of Animal Sciences, College of Food, Agricultural and Environmental Sciences, The Ohio State University, Wooster, OH 44691, USA; niu.214@osu.edu; 2 Department of Veterinary Preventive Medicine, College of Veterinary Medicine, The Ohio State University, Columbus, OH 43210, USA

**Keywords:** porcine epidemic diarrhea virus, live attenuated vaccines, recombination, safety issue

## Abstract

Porcine epidemic diarrhea (PED), causing up to 100% mortality in neonatal pigs, is a highly contagious enteric disease caused by PED virus (PEDV). The highly virulent genogroup 2 (G2) PEDV emerged in 2010 and has caused huge economic losses to the pork industry globally. It was first reported in the US in 2013, caused country-wide outbreaks, and posed tremendous hardship for many pork producers in 2013–2014. Vaccination of pregnant sows/gilts with live attenuated vaccines (LAVs) is the most effective strategy to induce lactogenic immunity in the sows/gilts and provide a passive protection via the colostrum and milk to suckling piglets against PED. However, there are still no safe and effective vaccines available after about one decade of endeavor. One of the biggest concerns is the potential reversion to virulence of an LAV in the field. In this review, we summarize the status and the major obstacles in PEDV LAV development. We also discuss the function of the transcriptional regulatory sequences in PEDV transcription, contributing to recombination, and possible strategies to prevent the reversion of LAVs. This article provides insights into the rational design of a promising LAV without safety issues.

## 1. Introduction

Porcine epidemic diarrhea virus (PEDV) is a major porcine enteric pathogen causing severe intestinal infection in neonatal pigs, resulting in acute diarrhea, vomiting, dehydration, and mortality. The disease, porcine epidemic diarrhea (PED), was initially discovered in the United Kingdom in 1971 and then spread to multiple pork-producing countries in Europe [1], when the pathogen behind it remained veiled. In 1978, PEDV was first identified as the causative agent for the disease by researchers from Belgium [2,3]. It caused widespread infections in European farms, leading to severe losses in suckling pigs in the 1970s and 1980s. Then, it became rare in Europe in the 1990s, with sporadic outbreaks in adult pigs or mild symptoms in suckling pigs. The first case of PED in Asia was reported in 1982 and outbreaks continued through the 1990s. Then, it became endemic until 2010, when the highly virulent PEDV strains emerged in China [4]. PEDV was introduced into the United States in 2013 and rapidly spread throughout the country [5,6,7,8]. During the 2013–2014 epidemic, over 50% of sow farms tracked in the Swine Health Monitoring Project had new outbreaks with an estimated USD 900 million to USD 1.8 billion economic loss [9,10]. PEDV is a member of the Alphacoronavirus genus in the Coronaviridae family of the Nidovirales order. It is one of the largest RNA viruses, carrying an approximately 28,000-nucleotide-long, positive-sense, single-stranded RNA genome with a 5′ cap and a 3′ polyadenylated tail [2]. Due to the large RNA genome, coronaviruses (CoVs) including PEDV has adapted to having a proofreading enzyme and can balance the conflict between replication fidelity and genetic diversity [11]. PEDV undergoes an evolutionary path by accumulating mutations and going through recombination events that enable increased viral fitness. Two large ORFs, ORF1a and ORF1b, are encoded at the 5′ two-thirds of the PEDV genome, followed by the ORFs for four structural proteins and one accessory protein: spike (S), ORF3, envelope (E), membrane (M), and nucleocapsid (N) proteins [12]. During viral replication, ORF1a and ORF1b are translated into two polyprotein precursors that are post-translationally further processed into 16 nonstructural proteins (nsp1–nsp16) by viral proteases. Among all proteins encoded by PEDV, the S glycoprotein forms the trimeric projections on the virion surface and is responsible for attachment to host receptors and mediates virus entry to the cell by membrane fusion, initiating the infection process. Moreover, the S gene is a hypervariable region among PEDV strains. Therefore, it serves as a phylogenetic marker for determining the genetic diversity of PEDV [13,14,15]. Based on genetic diversification of S proteins, PEDV can be genetically separated into two genogroups: genogroup 1 (G1) and genogroup 2 (G2), which can be further divided into subgroups G1a, G1b and G2a, G2b, and G2c [16,17,18]. PEDV G1a includes the prototype strain, CV777, identified in Belgium, and all strains sharing high genetic identity with CV777 [19,20]. The emerging highly virulent strains are classified as G2 [8,16,21]. Since a set of sub-genomic RNAs (sgRNAs) sharing identical 5′ end is synthesized during CoV replication, homologous recombination rates among different strains are relatively high in CoVs, including PEDV [22]. Consequently, the potential recombination events between the G1a and G2 strains led to the emergence of the S-INDEL strains, G1b, and one recently defined G2c [8,16,23,24], suggesting complex and rapid evolution of PEDV. Although herd immunity and biosecurity remain the most efficient ways for preventing PED, the constant emergence of new variants, including strains derived from recombination events, has led to vaccine failure, and hinders the prevention and control of PEDV [25,26]. This review focuses on the molecular basis of viral transcription, and the rational design of safe and effective live attenuated vaccines (LAVs) for PEDV.

## 2. PEDV Replication and Functional Elements in Transcription

The initial step of PEDV infection is recognizing and binding to host receptors through the S protein. The host receptor usages have been identified for several CoVs. Middle East respiratory syndrome coronavirus (MERS-CoV) recognizes dipeptidyl peptidase 4 (DPP4) for receptor binding [27,28]. Severe acute respiratory syndrome coronavirus (SARS-CoV) and SARS-CoV-2 bind to angiotensin-converting enzyme 2 (ACE2) to initiate infection [29,30]. While some CoVs, including transmissible gastroenteritis virus (TGEV) as well as its variants, porcine respiratory coronavirus (PRCV), porcine deltacoronavirus (PDCoV), human coronavirus (HCoV)-229E, and feline CoV type II, utilize aminopeptidase N (APN) as receptors [31,32,33,34]. APN is a 150 kDa transmembrane proteolytic enzyme that cleaves neutral or basic NH2-terminal residues from peptides [35]. It is a ubiquitously expressed protein on epithelium cells, macrophages, and granulocytes. APN is involved in extensive biological processes, including cell proliferation, motility, adhesion, and endocytosis [36,37]. Previous studies suggested APN as a co-receptor for PEDV infection. The S protein of PEDV can efficiently bind to both human and porcine APNs. Then, one non-permissive canine kidney cell line MDCK, exogenously expressing human or porcine APN (pAPN), could support PEDV infection and serial viral passaging [38,39]. Conversely, pretreatment of the susceptible and permissive cells with anti-pAPN antibodies blocked the productive infection of PEDV [39]. Meanwhile, PEDV replicated in a transgenic mouse model expressing pAPN [40]. However, because the APN-deficient cell line, Vero, and pAPN-knockout pigs supported PEDV replication, there must be unknown receptors for PEDV [41,42,43].

**Figure 1:**
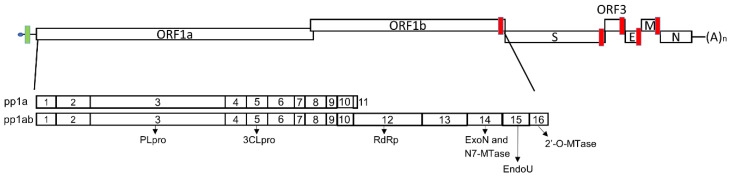
The genomic organization of a PEDV and its structural (S, E, M, and N), non-structural (nsp1–16), and accessory (ORF3) proteins. The green and red bars located at the 5′ UTR or upstream of each ORF represent the leader TRS and body TRS regions. Abbreviations: a number for pp1a and pp1ab indicate the non-structural proteins 1–16. PLpro: Papain-like protease; 3CLpro: chymotrypsin-like protease; RdRp: RNA-dependent RNA polymerase; ExoN: Exoribonuclease; N7-MTase: N7-methyltransferase; EndoU: endoribonuclease; 2′-O-MTase: 2′-O methyltransferase; S: spike; E: envelop; M: membrane; and N: nucleocapsid.

Following entry into host cells, the PEDV genome is released into the cytoplasm and gets access to the cellular machineries for viral replication. PEDV has a positive-sense RNA genome that serves as the mRNA for initial viral protein translation. The ORF1a and ORF1b encode two polyproteins, pp1a and pp1ab, which are polyprotein precursors and self-processed into 16 nonstructural proteins (nsps) by two viral proteases, the papain-like protease (PLpro) and the 3C-like protease (3CLpro) within nsp3 and nsp5, respectively (Figure 1). During viral replication, nsp1 is generated and released rapidly from the polyproteins upon translation [44]. It targets host translation machinery and the interferon (IFN) response system to induce host mRNA degradation and antagonize IFN responses [45,46,47,48]. The replication–transcription complex (RTC), responsible for viral RNA synthesis, consists of multiple viral nonstructural proteins [49,50,51]. Among the proteins involved in the RTC, nsp3–nsp6 are responsible for modulating intracellular membranes and assembling double-membrane vesicles (DMV), where viral RNA synthesis occurs [52,53]. Further, the remaining nsps contain the core enzymatic functions involved in RNA synthesis. For examples, heterodimers of nsp7 and nsp8 initiate the nascent RNA synthesis and generate short RNA primers for replication, forming the minimal core for the CoV replication complex with nsp12 [54,55]. Nsp12 is an RNA-dependent RNA polymerase (RdRp) for RNA synthesis within the replication complex. During the elongation of nascent RNA, the RNA-binding protein nsp9 and helicase nsp13 are also involved. Moreover, the bifunctional protein nsp14 has both 3′–5′ exonuclease (ExoN) and N7-methytransferase (MTase) activities [11,56]. The exonuclease domain is responsible for proofreading activity by removing mis-incorporated nucleotides during RNA elongation to keep high replication fidelity. Further, the N7-MTase domain, along with a 2′-O-MTase, nsp16, mediates the capping process of viral RNAs, with the involvement of nsp10 as a co-factor [57,58]. Moreover, nsp15, a uridine-specific endoribonuclease (EndoU) conserved across CoVs, processes viral dsRNA to evade the detection by host defense systems [59,60], contributing to immune evasion.

**Figure 2:**
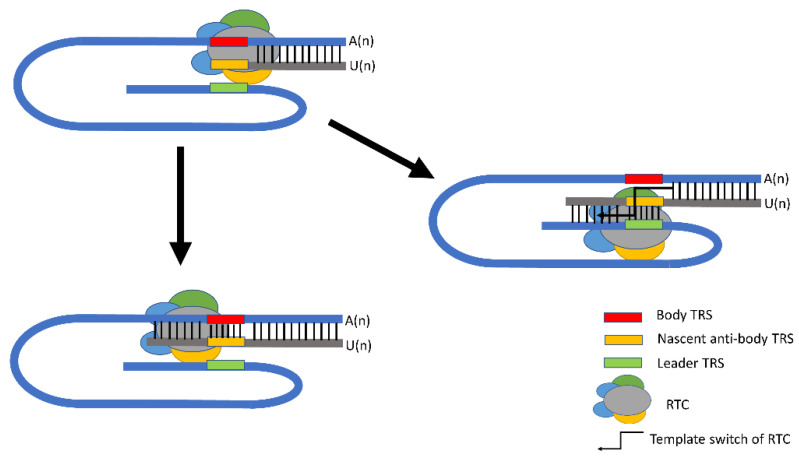
A discontinued replication model for PEDV. When RTC encounters TRS region, it will either “read through” or “template switch” to generate discontinued sgRNAs. Modified from Baker, S.C., 2008 [62].

Viral RNA synthesis following the initial translation and assembly of RTC includes viral genome replication and transcription. For viral genome replication, RTC can recognize the PEDV positive-sense RNA genome to copy it and produce a complimentary negative-sense genome continuously. Then, the negative-sense genomic copies serve as templates and more nascent positive-sense genomic RNAs are synthesized and eventually incorporated into progeny virions. Unlike the continuous genome replication, PEDV utilizes a discontinuous strategy for its transcription. There is a set of 5′ and 3′ co-terminal sub-genomic RNAs (sgRNAs) produced upon the discontinued mechanism during negative-sense RNA synthesis [61] and the negative-sense sgRNAs subsequently serve as the templates to produce positive-sense sub-genomic messenger RNAs (sgmRNAs). All sgmRNAs share an identical 5′ region, called a leader sequence, which is located at the beginning of the CoV genome, while during the synthesis of sgmRNAs, a set of cis-acting elements, called transcriptional regulatory sequences (TRS), is required. TRSs are short sequences with high homology and located upstream of each body ORF (called “body TRS”) and downstream of the 5′-leader sequence (called “leader TRS”) (Figure 1). In the process of negative-sense RNA synthesis, the elongation of the nascent strand is interrupted when RTC encounters body TRSs of N, M, E, ORF3, and S genes in the 3′ one-third of the viral genome. In this case, the body TRSs work as a “slow-down” or “stop” signal for RTC, which will either read through to transcribe the next ORF or switch the template to leader TRS, generating a sgRNA, carrying the reverse complementary sequence of the 5′ leader sequence (Figure 2).

The template switch event at body TRSs involves interaction between the reverse complementary TRSs (negative-sense body TRS or nascent anti-body TRS) of the nascent negative RNA strand and the 5′ UTR of genomic RNA (positive-sense leader TRS). Within the TRS region, there is a conserved core sequence (CS) that consists of 6 to 8 nucleotides and is flanked by variable sequences at its 5′- and 3′ ends. Based on systematic analysis, Yang et al. found that leader TRS-CS is conserved within a genus but different between genera, except embecoviruses in the *Betacoronavirus* genus, whose TRS-L CS is like that of alphacoronaviruses but not betacoronaviruses [63]. For example, all alphacoronaviruses, including TGEV, swine acute diarrhea syndrome coronavirus (SADS-CoV), and PEDV, share identical leader TRS-CS (5′-CUAAAC-3′). As for body TRS-CS, even viruses within the same genus show diversity. TGEV has nine highly conserved body TRS-CSs with the sequence of 5′-CUAAAC-3′, including one at the 5′ end of each ORF (1a, S, 3a, 3b, E, M, N, and 7) and an internal CS in the S gene [64]. For PEDV, the body TRS-CS of CV777 strain E, M, and N genes have been experimentally determined to be 5′-CUAGAC-3′, 5′-AUAAAC-3′, and 5′-CUAAAC-3′, respectively [65], and the putative TRS-CSs of S and ORF3 are 5′-GUAAAC-3′ and 5′-CCUUAC-3′, respectively. The body TRS-CSs can differ by up to three nucleotides from the leader TRS-CS in PEDV. Previous studies reported a critical role of the CS in guiding base pairing and duplex formation between nascent negative strands and the leader TRS site at the 5′ end of the genome [66,67]. In most cases, except internal initiations observed in murine hepatitis virus (MHV), infectious bronchitis virus (IBV), and bovine coronavirus (BCoV), only the 5′-most ORF within sgmRNAs is translated. Therefore, the homology of the leader-body TRS-CS was a key factor regulating sgmRNA transcription and subsequent translation [68]. Body TRS-CSs, governing the expression of different ORFs, exhibit diverse similarities to leader TRS-CS in PEDV and this may be another strategy to control the abundance of different sgmRNAs, as well as viral proteins.

**Figure 3:**
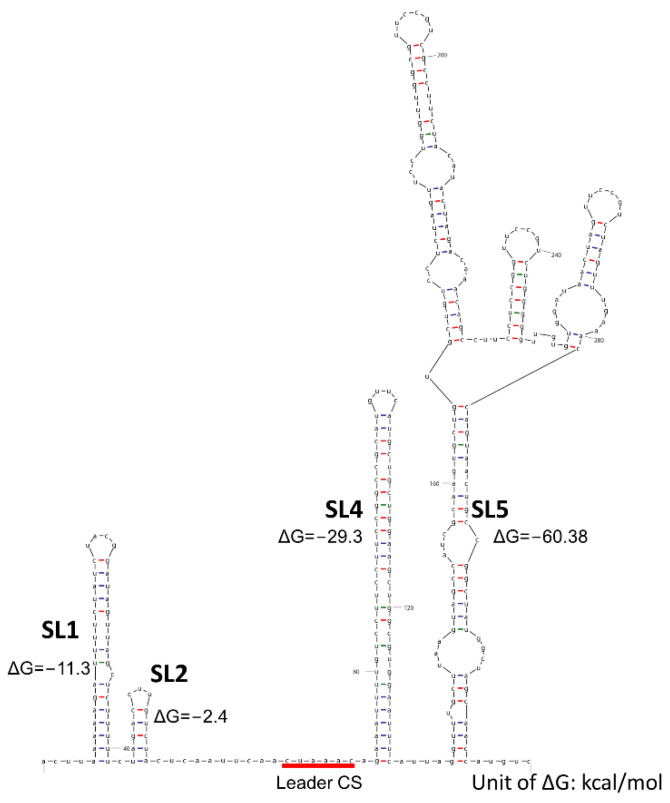
Secondary structures for the 5′ UTR of the prototype PEDV CV777 strain. Four conserved stem-loops (SLs) are predicted using Mfold [http://www.unafold.org/mfold/applications/rna-folding-form.php (accessed on 26 May 2022)], and Gibbs free energy for each SL was calculated and presented in kcal/mol.

In addition to the sequence similarity, RNA secondary structures are also considered as cis-acting elements required for RNA synthesis. The structural function of 5′ UTR in viral genome replication was first validated in a defective interfering RNA (DI RNA)-based system in BCoV. Four stem-loops (SLs), SL I, II, III, and IV, were predicted within BCoV 5′ UTR and mutation analysis suggested that those structures are essential for viral replication [69,70,71]. Later, three conserved SLs (SL1, SL2, and SL4) were identified from alphacoronaviruses (HCoV-NL63, HCoV-229E, and TGEV), betacoronaviruses (HCoV-OC43, HCoV-HKU1, SARS-CoV, and MHV), and gammacoronaviruses IBV [72,73]. MHV has served as a model to demonstrate that those conserved structures are critical for viral replication. SL1 was proposed to exist in an equilibrium with partially unfolded conformers. The structural destabilization of the SL1 by diminishing base pairing proved to be lethal or resulted in viruses with decreased replication, while compensatory mutations re-establishing the base pairing of SL1 restored viral replication to a similar level to wild-type virus [74]. Among the conserved SLs, SL2 has the highest consistency across all genera of CoVs. It has a U-turn motif, which may be responsible for RNA–RNA interactions. Mutagenesis analysis reported that mutations destabilizing the stem of SL2 significantly impaired MHV replication, resulting in decreased peak infectious titers and much smaller plaque sizes compared with wild-type MHV. Furthermore, the amount of RNA synthesized by the mutants with a destabilized SL2 was significantly lower than wild-type virus. Conversely, compensatory mutations that restored the base pairing can recover viral replication of those destabilized mutants to a comparable level with wild-type virus. In addition, mutants carrying transversion mutations, which disrupted the stem, were unviable [73]. It revealed that the SL2 is crucial for viral replication by regulating RNA synthesis. SL4 is a long hairpin structure located downstream of the leader TRS. It is proposed that the basal part of SL4 is in a flexible state that may be responsible for the establishment of transient long-range RNA–RNA interactions, leading to template switching during sgRNA synthesis [66]. Based on our analysis using Mfold [http://www.unafold.org/mfold/applications/rna-folding-form.php (accessed on 26 May 2022)], four secondary structures, SL1, SL2, SL4, and SL5, are predicted within the 5′ UTR of the PEDV CV777 strain (Figure 3). Besides the conserved SLs, an additional SL5 is mapped between nucleotide (nt) residues 123 and 305. It is a large structure containing three hairpin-loops that extend to ORF1a. To date, functions of these secondary structures adjacent to the leader TRS within the 5′ UTR remain veiled and further experiments are needed to confirm their role in the context of PEDV RNA replication.

## 3. Status of PEDV Vaccine Development

Productive infection will be initiated based on the collaboration of various viral proteins and functional elements within the genome and the infection triggers host systemic and local mucosal immune responses against viral infection. An earlier study suggested that the immune response induced by prior exposure protects against re-infection in weaned pigs [26]. In addition, PEDV-exposed gilts can passively transfer maternal immunity via the gut-mammary-gland-secretory IgA axis and provide the piglets up to 100% protection against PED after challenge [75]. These results suggested that viral-replication-induced host immunity, especially lactogenic immunity, is an effective way to prevent PED. Won et al. [76] and Lv [77] et al. reviewed PEDV vaccines, including LAVs, and inactivated, vectored, and subunit PEDV vaccines. Prior to 2010, the G1a PEDV-based vaccines, including inactivated and live-attenuated types, effectively controlled PEDV outbreaks in Asian countries. Ma et al. prepared one inactivated vaccine based on a cell-adapted CV777 strain in 1994, which can provide a protection rate of 85.19% in 3-day-old pigs, and a passive immunization protection rate of 85.0% in piglets when the sows were vaccinated [78]. Later, the same group successfully developed an inactivated bivalent TGEV and PEDV vaccine and made it commercially available in 1995 in China [79]. In addition to the inactivated vaccines, Tong et al. reported one LAV generated through serial passage of CV777 in vitro [80]. It exhibited a 95.52% protection rate and a 96.2% passive immunization protection rate in three- to six-day-old piglets [80]. In 1999, one bivalent LAV for PEDV and TGEV was successfully developed and provided active and passive protection rates against PEDV as high as 97.7 and 98%, respectively [81,82]. The two bivalent vaccines were widely used in China and effectively controlled the spread of PEDV and TGEV before the emergency of the highly virulent PEDV variants in 2010. Vaccines have also been developed by Japan and South Korea. One Japanese strain, PEDV 83P-5, was attenuated upon serial passages in Vero cells and is commercially available as an LAV [83]. Importantly, the 83P-5 inoculation of sows passively protected 80% of piglets from death against G2 PEDV challenge [84]. Two South Korean virulent strains SM98-1 and DR-13 were also passaged in vitro and attenuated. The SM98-1 strain has been used as an intramuscularly administrated LAV or inactivated vaccine, and the DR-13 strain is available as an oral LAV [85,86]. Song et al. showed that orally administration of DR-13 to late-term pregnant sows passively protected 87% of suckling piglets after homologous challenge [86].

Since late 2010, China experienced severe PED outbreaks with devastating damage to the swine industry due to the emergence of highly virulent PEDV variants falling into the G2 branch [4], which spread to other Asian and North American countries and to Europe (Ukraine) [87,88]. Two multivalent vaccines were officially approved and launched on the market in China in 2015 [89]. One is a trivalent vaccine developed from attenuated TEGV, PEDV (CV777 strain), and porcine rotavirus and a bivalent attenuated vaccine containing TGEV and PEDV (ZJ08 strain, G1b), but their efficacy is questionable for the poor cross-protection between the G1 and G2 strains. A comparative study evaluated the efficacy of the G1b- and G2b-based vaccines against G2b strain challenge in 2-week-old weaned pigs. It suggested that an inactivated G2b-based vaccine provided sufficient protection against G2b challenge, as evidenced by a reduction in PEDV RNA in feces for 3–4 logs during peak shedding and a shorter viral shedding duration, but the G1b strain-derived vaccine failed [90]. A similar phenomenon was observed from South Korea and Thailand, where commercially available vaccines, derived from G1 strains, failed to provide complete protection against the currently circulating strains belonging to the G2 group [4,88,91,92]. To date, the highly virulent PEDV is one of the major swine viral pathogens in China (containing > 50% of the world’s pig population) [93,94]. Therefore, effective vaccines targeting G2 strains are in urgent need to prevent and control this deadly viral infection in piglets.

Most of the licensed PEDV vaccines are inactivated or LAVs consisting of entire pathogens that have been killed or fully attenuated. Such whole-pathogen vaccines can elicit strong protective immune responses. Collin et al. developed an inactivated vaccine based on the isolated US variant NPL-PEDV 2013 P10.1 strain, belonging to G2b [95]. The vaccine elicited a considerable level of humoral immunity against PEDV through intramuscular administration, according to cell-based viral neutralization assays. However, inactivated vaccines do not replicate and can only induce a less-broad immune response. Furthermore, the immunity induced by inactivated vaccines is not as long lasting as LAVs, leading to requirements for multiple doses for boosting. Further, strong passive lactogenic immunity triggered by LAVs is the most promising and effective way to protect neonatal suckling piglets from enteric diseases, including PEDV [96].

Serial passaging in non-natural host tissue culture leading to attenuation is a conventional approach to develop LAVs. To date, several cell-attenuated G2 strains have been reported, including the US isolate PC22A, Asian strains YN, Pingtung-52, and KNU-141112 [97,98,99,100]. Hou et al. thoroughly reviewed the mutation patterns and molecular mechanisms of the four attenuated strains [101]. In general, the cell-adapted strains are attenuated in piglets and highly immunogenic by eliciting a high level of neutralizing antibodies.

However, there are several concerns about protective efficacies of these reported G2 PEDV-based LAV candidates. First, as the most vulnerable population is sucking pigs and there is no enough time to induce active immunity in them before they encounter the virus, the ideal strategy for PEDV vaccination is inducing protective lactogenic immune responses in sows/gilts. Since PEDV-challenge study in sows/gilts is costly and labor intensive, scientists utilize cell-based viral neutralization assays to test the protective immune responses induced by vaccination, which may serve as an indicator for protection. Additionally, a neonatal pig model is used in testing virus attenuation and nursery (or weaned) pigs are used to evaluate immunogenicity of viruses and screen promising candidates. For example, in the study of the cell-culture-adapted PC22A strain, viruses at the 100th passage (P100) and P120 were fully attenuated in weaned pigs, but partially attenuated in neonatal piglets. However, the P100 of PC22A induced better titers of serum PEDV IgA, IgG, and viral neutralization (VN) antibodies and higher numbers of PEDV IgA antibody-secreting cells after the virulent strain challenge than the P120 virus [102]. These results suggest that attenuation of PEDV is a double-edged sword. LAV candidates that were complete attenuated in piglets could not induce enough lactogenic immunity in sows. Older pigs are more resistant to PEDV infection and disease than piglets [103]. Therefore, the full attenuation of a PEDV in piglets often causes inefficient replication of the attenuated virus and decreased viral immunogenicity in older pigs, resulting in inefficient protective immunity. Secondly, low cross-protection between G1 and G2 groups has been described before, and the cross-protective efficiency between G2a and G2b is also a concern in vaccine development. Liu et al. demonstrated that both CH/HBXT/2018 (G2a) and CH/HNPJ/2017 (G2b)-inactivated vaccines induced significantly lower VN antibody titers against heterologous strains than homologous strains [104]. Thus, challenge with heterologous strains is important to elucidate cross protection in further vaccine studies.

## 4. Risk and Prevention of Virulence Reversion of PEDV Live Attenuated Vaccines

In addition to the protection efficiency, one of the biggest concerns hindering the application of PEDV LAVs is the safety issue. The attenuated strains carry mutations, introduced by serial passage or molecular engineering, making them devoid of pathogenicity. However, some vaccine strains exhibit reversion to virulence during passage in the primary vaccine recipient through (1) accumulation of mutations within the viral genome, and (2) recombination. In this section, we will review strategies counteracting the reversion events for PEDV LAV development.

There are two approaches to generate promising attenuated vaccine candidates: (1) the classical approach by serial passaging viruses in a non-natural host or environment, leading to adaptation in the new conditions and attenuated replication in the natural host, or (2) the reverse genetics approach by genetically modifying a variety of genes that are dispensable for viral viability for decreased pathogenicity. However, due to the immunocompromised conditions of hosts or the genetic instability for less-vigorous viruses, both reversion of attenuating mutations and compensatory mutations elsewhere in the genome may lead to virulence reversion. The 2′-O-MTase of nsp16 is highly conserved among CoVs and mediates the capping process of viral genomic RNA and sgmRNAs during replication and transcription. One SARS-CoV nsp16 mutant (dNSP16) demonstrated efficacy as a vaccine candidate following heterologous challenge in an aged mice model. However, in a mice model lacking functional B and T cells (RAG−/−), following inoculation with the dNSP16 mutant, 62.5% (5/8) of the immunocompromised mice showed weight loss and lethality, which was absent in the aged mice model, suggesting a reversion to virulence [105]. Meanwhile, although the introduced mutation targeting nsp16 was retained in the revertant, six mutations were found in nsp3, nsp12, and nsp15, which may potentially function as compensatory mutations. A previous study demonstrated that one PEDV nsp14-ExoN mutant, E191A, was significantly attenuated but of high genetic instability, and back mutations were observed both in vitro and in vivo [11]. Similar stories were reported for the recombinant MHV and SARS-CoV carrying disrupted nsp14 ExoN domains. Upon serial passage in vitro (250 times), although no back-mutations were found at the ExoN(-) active site, the MHV mutant with engineered ExoN (MHV-ExoN(-)-P250) accumulated eight-fold-more mutations than wild-type MHV and demonstrated increased replication fidelity, suggesting the emergence of compensatory mutations for ExoN function during viral replication [106].

To mitigate this mutation-driven reversion, multiple mutations targeting separate genes and attenuating the virus via distinct mechanisms can be combined into the viral genome. For example, we generated a recombinant PEDV icPC22A-KDKE4A-SYA carrying inactivated nsp16 2′-O-MTase and the endocytosis signal of S protein [58]. As mentioned above, dysfunction of nsp16 2′-O-MTase attenuated SARS-CoV [105,107], MHV [108], and MERS-CoV in mice [109]. The conserved motif YxxΦ at the cytoplasmic tail of the S protein regulates the level of S proteins on the infected cell surface and functions as a virulence factor [110]. The recombinant mutant icPC22A-KDKE4A-SYA retained the introduced mutations after passaging three times in pigs, indicating its genetic stability in vivo. A similar approach counteracting reversion was used in SARS-CoV LAV development by combining inactivated nsp14-ExoN and nsp16-2′-O MTase to generate dNSP16/ExoN [105]. Unlike the dNSP16 mutant, dNSP16/ExoN did not cause significant diseases and was cleared in the immunocompromised mice model without reverting to a virulent form after 30 days post inoculation. Collectively, the combination of multiple mutations to attenuate a virus through various pathways provides an answer to combat mutation-driven reversion of CoV LAVs.

Furthermore, recombination is an important evolutionary factor for many RNA viruses, especially CoVs [111] that have high recombination rates approaching 20% during a mixed infection of closely related strains [112]. Recombination-driven reversion, when a vaccine strain recombines with field-virulent strains giving novel variants, poses an obstacle to the application of LAVs. Vaccine failures caused by recombination-driven reversion occurred for several LAVs for animal viruses, including canine parvovirus [113], infectious bursal disease virus [114], bovine herpesvirus 1 [115], as well as members from CoVs, IBV [116], and PEDV [117]. PEDV-recombinant variants were observed from several major pig-farming provinces in China [117,118,119]. One variant exhibiting high pathogenicity in the field was derived from a recombination event between low-pathogenic vaccine and virulent field strains [117]. Virus genomes recombine by one of three general mechanisms: (i) break and repair in DNA genomes, (ii) polymerase template switching in RNA genomes, and (iii) reassortment of segments in segmented RNA genomes [120]. As a non-segmented RNA virus, CoVs readily perform both inter-molecular recombination between two distinct molecules and intra-molecular recombination within the same molecule via template switching. The intra-molecular recombination is defined when the replicase switches between the leader and body TRS regions, generating a set of sgmRNAs, while the inter-molecular recombination occasionally gives recombinant virus progenies when replicase jumps from donor to acceptor templates of different parent strains, sharing homologous sequences during RNA synthesis. Systematic analysis for TRS sites and recombination events in CoVs suggested that nearly 10% body TRS regions are involved in breakpoint hotspots and the recombination hotspots are frequently correlated with body TRSs [62,121]. Thus, the TRS circuit becomes a main target to disable the inter-molecular recombination during CoVs replication. One recombination-resistant SARS-CoV was engineered by the introduction of 3-nt into the rewired TRS that differed from the wild-type TRS sequence at three nucleotides [122]. The rewired TRS circuit was incompatible with the wild-type one and was responsible for the failure of rescue chimeric viruses carrying mixed wild-type and rewired TRSs. Later, researchers from the same group further optimized the regulatory circuit and designed a 7-nt rewired TRS that showed enhanced genetic stability and the genome can serve as an effective recombination-resistant platform for SARS-CoV-attenuated vaccine development [123].

For PEDV, there are two difficulties in rewiring the TRS circuit: (1) unlike the high identity of TRS-CSs observed in most CoVs, the TRS-CSs of PEDV exhibited incredible diversity, among which the body TRS-CSs can differ by three nucleotides from the leader TRS-CS (described in Section 2); (2) within the PEDV genome, all the body TRS-CSs overlap with the upstream ORFs. Therefore, the introduction of mutations into the TRS regions may alter amino acid sequences in the upstream ORFs, leading to unfavored mutations. New approaches instead of directly recoding TRS-CSs are needed to prevent TRS-related recombination-driven reversion. The first possible approach is re-designing the TRS-CSs overlapping with upstream ORFs by introducing silent mutations to retain the original amino acid sequences. Besides silent mutations, a conservative amino acid replacement strategy, in which an amino acid in a protein is replaced by another amino acid with similar biochemical properties, can also be employed to design TRS-CSs. In addition to the aspect of sequence similarity, RNA secondary structures of the TRS regions are considered as cis-acting elements, regulating RNA transcription as well. As we discussed in Section 2, the secondary structure of the 5′ UTR of PEDV genome plays an important role in viral replication. However, limited information is available to accurately predict the secondary structure, especially for the body TRSs. Researchers reported dynamics of secondary structures of a CoV genome during its life cycle according to SARS-CoV-2 [124]. For example, an RNA genome within virion undergoes major conformation alteration and shows intensive compaction compared to the viral RNAs in the infected cells. Although the secondary structure of 5′ UTR of the PEDV CV777 strain was predicted (Figure 3), more approaches to recode the PEDV TRS system by targeting the critical structural elements within the body TRS regions will benefit from illustration of the dynamics of PEDV genome structures during viral replication. Structural changes in the regions may also modulate the template switch event of RTC, leading to an altered transcription circuit. Using these approaches, the recoded body TRS-CSs will be incompatible with wild-type ones but retain the conservative substitutions of upstream ORFs. At last, we can disrupt the original TRS sites and reorganize the genome of PEDV by artificially introducing gaps to separate the TRS regions from the upstream ORF. Thus, the introduced gaps are expected to serve as functional elements regulating PEDV transcription and we can rewire the transcription circuit as previously described in SARS-CoV [122,123]. Currently, we are working on the design of a remodeled TRS that barely changes the secondary structure of the original 5′ UTR. A remodeled PEDV mutant RMT has been rescued that replicated efficiently in vitro and in vivo. The RMT showed a partially attenuated phenotype and induced partial protection in neonatal piglets (unpublished data). It also showed decreased recombination with a wild-type S-INDEL PEDV strain. Thus, it can be used as a platform to develop recombination-resistant PEDV LAVs in the future.

## 5. Conclusions

The emerging highly virulent PEDV caused massive outbreaks with high mortality in suckling piglets, leading to major losses to the pork industry, but few vaccines provide effective protection against the disease. Because of the vulnerability of newborn pigs, the most effective vaccination strategy is inducing strong lactogenic immunity in pregnant sows, which can passively transfer the protective neutralizing antibodies to suckling piglets via colostrum and milk. Meanwhile, active mucosal immunity protecting the gut of sows is critical in that process [96]. Previous studies on another porcine enteric coronavirus, TGEV, showed that only immunization with live virus, but not inactivated or subunit vaccines, triggered sufficient lactogenic immunity [125]. Therefore, LAVs for use in sows readily triggering lactogenic immunity are a promising approach for the prevention and control of PED. In the future, homologous and heterologous challenging is needed to show cross-protection of the vaccines against field-circulating strains. Additionally, the safety issue for virulent revertants of attenuated strains remains unsolved and hinders the application of LAVs. Using reverse genetics and newly developed approaches, the combination of several attenuation mutations that do not decrease the viral immunogenicity (e.g., mutations in nsp1) and rewired TRSs, can help increase genetic stability of the vaccine candidates that may become more resistant to mutation- and recombination-driven reversion.

## Data Availability

Not applicable.
